# Experiments Regarding Anthrax

**Published:** 1881-01

**Authors:** 


THE EXPERIMENTS REGARDING ANTHRAX.—The experiments referred to regarding splenic fever were made by Burdon-Sanderson and Greenfield upon heifers suffering from that disease. The blood from a heifer with splenic fever was inoculated in a guinea-pig. This animal took the disease. Its blood was then injected into a healthy heifer. A slight febrile reaction followed, after which it was found that the animal was no longer susceptible to the poison of the splenic fever.

The experiments of Toussaint upon anthrax in sheep were conducted as follows:

The blood taken from a diseased animal was raised to a temperature of 131° F., and one-half of one per cent. of carbolic acid added. The mixture was then allowed to stand for two or three days, until the *bacilli* were all destroyed. About a drachm of the fluid was then injected subcutaneously into a sheep. A slight fever followed, which lasted a few days. In ten or twelve days, the animal was found to be "protected," and, when inoculated with fresh anthrax blood, was not in the least affected by it.

It may be observed of these two series of experiments, that they both tend to show a possible value for inoculation. But neither of them gives any strength to the bacterial theory of anthrax—the experiments of Toussaint, indeed, pointing directly against it. We may yet have to return to the view of Brauell, who discovered the *bacillus anthracis*, but refused to believe in its causative relation to anthrax.

